# Cooperative Interactions between TLR4 and TLR9 Regulate Interleukin 23 and 17 Production in a Murine Model of Gram Negative Bacterial Pneumonia

**DOI:** 10.1371/journal.pone.0009896

**Published:** 2010-03-26

**Authors:** Urvashi Bhan, Megan N. Ballinger, Xianying Zeng, Michael J. Newstead, Matthew D. Cornicelli, Theodore J. Standiford

**Affiliations:** Division of Pulmonary and Critical Care Medicine, Department of Internal Medicine, University of Michigan Medical Center, Ann Arbor, Michigan, United States of America; New York University, United States of America

## Abstract

Toll like receptors play an important role in lung host defense against bacterial pathogens. In this study, we investigated independent and cooperative functions of TLR4 and TLR9 in microbial clearance and systemic dissemination during Gram-negative bacterial pneumonia. To access these responses, wildtype Balb/c mice, mice with defective TLR4 signaling (TLR4^lps-d^), mice deficient in TLR9 (TLR9^−/−^) and TLR4/9 double mutant mice (TLR4^lps-d^/TLR9^−/−^) were challenged with *K. pneumoniae*, then time-dependent lung bacterial clearance and systemic dissemination determined. We found impaired lung bacterial clearance in TLR4 and TLR9 single mutant mice, whereas the greatest impairment in clearance was observed in TLR4^lps-d^/TLR9^−/−^ double mutant mice. Early lung expression of TNF-α, IL-12, and chemokines was TLR4 dependent, while IFN-γ production and the later expression of TNF-α and IL-12 was dependent on TLR9. Classical activation of lung macrophages and maximal induction of IL-23 and IL-17 required both TLR4 and TLR9. Finally, the i.t. instillation of IL-17 partially restored anti-bacterial immunity in TLR4^lps-d^/TLR9^−/−^ double mutant mice. In conclusion, our studies indicate that TLR4 and TLR9 have both non-redundant and cooperative roles in lung innate responses during Gram-negative bacterial pneumonia and are both critical for IL-17 driven antibacterial host response.

## Introduction

Pneumonia is a leading infectious cause of mortality in immunocompetent individuals in the United States. *Klebsiella pneumoniae* is a Gram negative bacteria that is a well described cause of both community acquired and hospital acquired pneumonia [1]. Mortality in pneumonia caused by *K. pneumoniae* is due to propensity for early systemic bacterial dissemination resulting in sepsis, and the development of acute lung injury [2]. Early clearance of the pathogen from the lung is required to prevent *Klebsiella* pneumonia associated complications [3].

Toll like receptors (TLRs) are a family of type I transmembrane receptor proteins that are required for the recognition of various pathogen-associated molecular patterns expressed by a diverse group of infectious microorganisms, resulting in the activation of host immune responses [4]. For example, TLR4 has been shown to be required for effective innate immunity against selected extracellular Gram-negative pathogens, including *Haemophilus influenza, Eschericia coli* and *Klebsiella pneumoniae* [5], [6], [7]. However, although innate signals produced early (at 4 h) in response to challenge with *K. pneumoniae* are markedly diminished in mice with defective TLR4 signaling, later responses (at 16 h) remain largely intact [5]. TLR9 has also been shown to be important for innate host defense against Gram-negative bacteria, including *Klebsiella* and *Neisseria* [8], [9]. Mice lacking TLR9 display impaired bacterial clearance when challenged with *K. pneumoniae* i.t., which is associated with reduced dendritic cell recruitment and activation, decreased type 1 cytokine expression, and alternative rather than classical activation of lung macrophages [8]. Importantly, host innate responses against both extracellular and intracellular bacterial pathogens are more dramatically impaired in mice that lack the common adaptor molecule MyD88 than in mice that are deficient in a single TLR (e.g., TLR2 or TLR4), suggesting cooperativity of various TLRs or the involvement of other MyD88-dependent TLRs [10], [11]. Collectively, these data indicate that multiple MyD88-dependent TLRs are required for the maintenance and/or full expression of protective innate responses during Gram-negative bacterial pneumonia.

To further investigate potential interactive role of TLRs in generating host defense in the lung during Gram-negative infection, we assessed innate antibacterial responses to i.t. *K. pneumoniae* challenge in mice with defective TLR4 signaling (TLR4^lps-d^), mice deficient in TLR9 (TLR9^−/−^) and double mutant mice (TLR4^lps-d^/TLR9^−/−^). We observed that maximal classical activation of lung macrophages, expression of IL-23 and IL-17, and lung bacterial clearance required cooperative interactions between TLR4 and TLR9, whereas survival responses in mice challenged with *K. pneumoniae* were largely dictated by the presence or absence of functional TLR4.

## Materials and Methods

### Reagents

Murine recombinant IL-17A for i.t. reconstitution experiments was purchased from R&D Systems (Minneapolis, MN).

### Mice

Female Balb/c (National Cancer Institute-Harlan, Frederick, MD) were used at 8 to 12 weeks of age. Breeding pairs of TLR9^−/−^ mice generated by S. Akira (Osaka, Japan) were obtained from Coley Pharmaceutical (Wellesley, MA) and a colony established at the University of Michigan. These mice were generated on a Balb/c background (>8 backcrosses), are phenotypically normal in the uninfected state, and reproduce without difficulty. Breeding pairs of mice with defective TLR4 signaling and bred onto a Balb/c background (TLR4^lps-d^) were obtained from Jackson Laboratories and a breeding colony established. TLR4^lps-d^ mice were then crossed with TLR9^−/−^ to generate TLR4^lps-d^/TLR9^−/−^ double mutant mice. All animals were handled in strict accordance with good animal practice as defined by the relevant national and/or local animal welfare bodies, and all animal work was approved by the UCUCA(University Committee on Use and Care of Animals) committee at the University of Michigan.

### K. pneumoniae inoculation

*K. pneumoniae* strain 43816 serotype 2 (American Type Culture Collection) was used in our studies. *K. pneumoniae* was grown overnight in tryptic soy broth (Difco) at 37°C and quantitated using spectrophotometry [12]. For i.t. administration, mice were anesthetized with an i.p. ketamine and xylazine mixture. Next, the trachea was exposed and 30 µl of inoculum was administered via a sterile 26-gauge needle. The skin incision was closed using surgical staples.

### Lung harvesting for bacterial number, and cytokine analyses

At designated time points, mice were euthanized by CO_2_ asphyxia. Prior to lung removal, the pulmonary vasculature was perfused with 1 ml of phosphate-buffered saline (PBS) containing 5 mM EDTA via the right ventricle. Whole lungs were then harvested for assessment of bacterial CFU and cytokine protein expression. After removal, lungs were homogenized in 1 ml of PBS with protease inhibitor (Boehringer Mannheim Biochemicals, Indianapolis, Ind.) using a tissue homogenizer (Biospec Products, Inc.) under a vented hood. Aliquots of homogenates (10 µl) were inoculated on nutrient agar after serial 1∶10 dilutions with PBS. The homogenates were incubated on ice for 30 min and then centrifuged at 1,100×*g* for 10 min. Supernatants were collected, passed through a 0.45-µm-pore-size filter (Gelman Sciences, Ann Arbor, Mich.), and stored at 20°C for assessment of cytokine levels.

### Bronchoalveolar lavage

Bronchoalveolar lavage (BAL) was performed at various time points post i.t. administration of bacteria. Briefly, the trachea was exposed and intubated using a 1.7-mm outer diameter polyethylene catheter. BAL was performed by instilling PBS containing 5 mM EDTA in 1 ml aliquots for a total of 3 mls. Lavaged cells were counted, cytospins performed and alveolar macrophages were harvested after adherence purification and cultured for 1 hour. Supernatants were collected and analyzed by ELISA for cytokine production and the cells were harvested for mRNA expression by real time PCR.

### Total lung leukocyte preparation

Lungs were removed from euthanized animals, and leukocytes were prepared as previously described [6], [11]. Briefly, lungs were minced with scissors to a fine slurry in 15 ml of digestion buffer [RPMI medium/10% fetal calf serum/1 mg/ml collagenase (Boehringer Mannheim Biochemical)/30 µg/ml DNase (Sigma)] per lung. Lung slurries were enzymatically digested for 30 min at 37°C. Any undigested fragments were further dispersed by drawing the solution up and down through the bore of a 10-ml syringe. The total lung cell suspension was pelleted, resuspended, and spun through a 40% Percoll gradient to enrich for leukocytes. Cell counts and viability were determined using trypan blue exclusion counting on a hemacytometer. Cytospin slides were prepared and stained with a modified Wright-Giemsa stain.

### Flow cytometry for intracellular IL-17 expression

Cells were isolated from lung digests as described above. For analysis of T cell subsets, isolated leukocytes were stained with the following FITC- or PE-labeled anti-γδ TCR anti-CD4 (BD Pharmingen). In addition, cells were stained with anti-CD45-tricolor (Caltag Laboratories), allowing for the discrimination of leukocytes from nonleukocytes and thus eliminating any nonspecific binding of T cell surface markers on nonleukocytes. Cells were then fixed and permeabilized using BD cytofix/cytoperm fixation/permeabilization kit for 20 min on ice. After washing, cells were stained for intracytoplasmic IL-17A expression with PE conjugated rat anti-mouse IL-17A Ab (BD Pharmingen) diluted in wash solution for 30 min. T cell subsets were analyzed by first gating on CD45-positive “lymphocyte-sized” leukocytes and then examined for FL1 and FL2 fluorescence expression using three color flow cytometry. Cells were collected on a FACScan or FACScalibur cytometer (BD Biosciences) by using CellQuest software (Becton Dickinson). Analyses of data were performed using the CellQuest software package.

### Isolation and culture of bone marrow-derived dendritic cells

Bone marrow was harvested from the long bones of mice using a previously described technique[8]. Recovered marrow cells were seeded in tissue culture flasks in RPMI 1640 based complete media with murine GM-CSF (10 ng/ml). Media and cytokines were replaced after 3 days, loosely adherent cells collected after 6–7 days and cells positively selected for CD11c+ by magnetic bead separation. CD11c+ DC were plated overnight and resuspended in fresh media the following day. Flow cytometry of cells verified >90% purity for DC. BMDC were cultured at a concentration of 5×10^5^ cells/ml, incubated with vehicle or heat-killed *K. pneumoniae* (10∶1 MOI), then supernatants harvested 16 hrs later.

### Expression of iNOS and Fizz-1 by alveolar macrophages

To assess spontaneous inducible nitric oxide synthase (iNOS) and Fizz-1 expression in alveolar macrophages, cells were isolated from lungs of WT and mutant mice post i.t. *Klebsiella* challenge by BAL and alveolar macrophages isolated by adherence purification and cultured for 1 hour at a concentration of 1−2×10^6^ cells/well. Cells were washed X 3, then RNA immediately isolated.

### Murine cytokine ELISAs

Murine TNF-α, KC/CXCL1, MIP-2/CXCL2, IL-12 p70, IFN-γ, IL-23 and IL-17 (R&D Systems, Minneapolis, MN) were quantitated using a modification of a double-ligand method as previously described [6], [11]. The ELISA method used consistently detected murine cytokine concentrations above 20–50 pg/ml. The ELISAs did not cross-react with other cytokines tested. [13]

### Real-time quantitative RT-PCR

Measurement of gene expression was performed utilizing the ABI Prism 7000 sequence detection system (Applied Biosystems, Foster City, CA) as previously described [6]. Primers and probe nucleotide sequences for miNOS, forward 5′- CCC TCC TGA TCT TGT GTT GGA-3′, reverse 5′-CAA CCC GAG CTC CTG GAA-3′, and probe 5′-TGA CCA TGG AGC ATC CCA AGT ACG AGT-3′; for m-actin: forward 5′-CCG-TGA-AAA-GAT-GAC-CCA-GAT-C-3′, reverse 5′-CAC-AGC-CTG-GAT-GGC-TAC-GT-3′, probe 5′-TTT-GAG-ACC-TTC-AAC-ACC-CCA-GCC-A-3′, for Fizz-1 forward 5′- CCC TGC TGG GAT GAC TGC TA-3′, reverse 5′-TCC ACT CTG GAT CTC CCA AGA -3′ and probe 5′- TGG GTG TGC TTG TGG CTT TGC -3′. Specific thermal cycling parameters used with the TaqMan One-Step RT-PCR Master Mix Reagents kit included 30 min at 48°C, 10 min at 95°C, and 40 cycles involving denaturation at 95°C for 15 seconds, annealing/extension at 60°C for 1 min. Relative quantification of cytokine mRNA levels was plotted as fold-change compared to untreated control lung. All experiments were performed in duplicate.

### Statistical analysis

Survival curves were compared using the log-rank test. For other data, statistical significance was determined using the unpaired t test or one-way ANOVA corrected for multiple comparisons as appropriate. All calculations were performed using the Prism 3.0 software program for Windows (GraphPad Software). All mean data shown are expressed as means ± SEM.

## Results

### Survival in WT, TLR4^lps-d^, TLR9^−/−^ and TLR4^lps-d^/TLR9^−/−^ double mutant mice after *K. pneumoniae* administration

**Figure 1:**
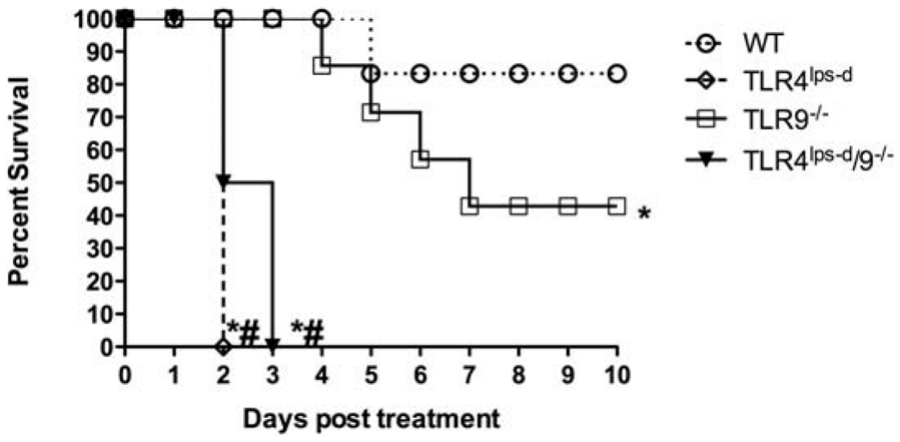
Survival after i.t. *Klebsiella* challenge in WT, TLR4^lps-d^, TLR9^−/−^, and TLR4^lps-d^/TLR9^−/−^ mice. n = 12 in each group. *p<0.01 as compared to WT mice. #p<0.01 as compared to TLR9^−/−^ mice. Combined from 2 separate experiments.

We and others have previously shown that both TLR4 and TLR9 play a critical role in host defense during *Klebsiella* pneumonia [5], [8], [14]. While the role of individual TLRs has been studied, the relative contribution and potential interactions between TLRs is unknown. For that reason, we administered *K. pneumoniae* 8×10^2^ CFU i.t. to WT, TLR4^lps-d^, TLR9^−/−^ and TLR4^lps-d^/TLR9^−/−^ double mutant mice, then assessed survival out to 10 days. As shown in Figure 1, TLR9^−/−^ mutant mice died more quickly and had reduced long term survival, as compared to WT mice (45% vs 85%, p<0.05). More impressively, all TLR4^lps-d^ single mutant and TLR4^lps-d^/TLR9^−/−^ double mutant mice died, with mortality observed as early as 48 hrs post *Klebsiella* administration and no animals surviving past 3 days.

### Bacterial clearance in WT, TLR4^lps-d^, TLR9^−/−^ and TLR4^lps-d^/TLR9^−/−^ double mutant mice after *K. pneumoniae* administration

**Figure 2:**
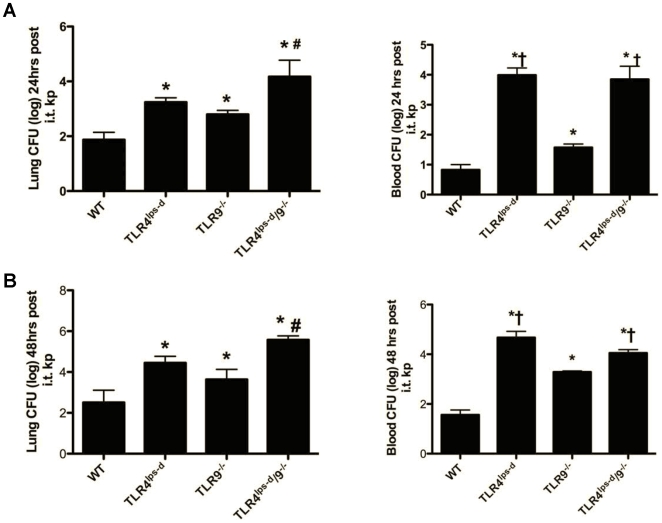
Lung and blood CFU from WT, TLR4^lps-d^, TLR9^−/−^, and TLR4^lps-d^/TLR9^−/−^ mice 24 hrs and 48 hrs post i.t. *Klebsiella* challenge. Mice were given 5×10^2^ CFU *Klebsiella* i.t., lungs and blood harvested at designated time points and CFU assessed. Panel A shows lung and blood CFU at 24 hrs and panel B is lung and blood CFU at 48 hrs post i.t. *Klebsiella* challenge. CFU are expressed in mean log_10_ ± SEM. n = 6−8, * p<0.05 as compared to WT mice, # p<0.01 as compared to single mutants, †<0.05 as compared to TLR9^−/−^ mice.

Having observed decreased survival in single and double mutants challenged with i.t. *Klebsiella*, we next explored the mechanism accounting for reduced survival. WT, TLR4^lps-d^, TLR9^−/−^ and TLR4^lps-d^/TLR9^−/−^ double mutant mice were challenged with 5×10^2^ CFU i.t. *K. pneumoniae* i.t., then lungs and blood harvested 6, 24 or 48 hours later. At 24 hours, we found that both TLR4^lps-d^ and TLR9^−/−^ mice challenged with *K. pneumoniae* i.t. displayed evidence of impaired lung bacterial clearance, as compared to WT mice [90 and 65 fold increase in CFU over WT, respectively, (Figure 2A, p<0.05)]. Double mutant (TLR4^lps-d^/TLR9^−/−^) mice had an even greater defect in lung bacterial clearance, as compared to single mutant animals (p<0.05), with a 300-fold increase in CFU as compared to infected WT animals. No bacteremia was observed in any group by 6 hrs post bacterial challenge. However, by 24 hrs bacteremia was observed in all mutant mice, with blood CFU greatest in TLR4^lps-d^ single mutant and TLR4^lps-d^/TLR9^−/−^ double mice. By 48 hrs (Figure 2B), all mutant mice (TLR9^−/−^, TLR4^lps-d^, TLR4^lps-d^/TLR9^−/−^) had statistically higher lung bacterial burden as compared to the WT mice, (53, 309, and 560 fold increase in CFU over WT mice, respectively, p<0.05). Mutant mice also had higher bacterial counts in blood, with the TLR4^lps-d^ and TLR4^lps-d^/TLR9^−/−^ double mutant mice having the highest bacteremic burden (704-fold and 523-fold, respectively, as compared to the WT mice). The TLR9^−/−^ also had statistically higher blood CFU (44-fold increase, as compared to the WT mice).

### Lung inflammatory cell influx in WT, TLR4^lps-d^, TLR9^−/−^ and TLR4^lps-d^/TLR9^−/−^ double mutant mice after *K. pneumoniae* administration

To assess the role of TLRs in lung leukocyte influx, we quantitated inflammatory cells in the lung digests of WT, TLR4^lps-d^, TLR9^−/−^ and TLR4^lps-d^/TLR9^−/−^ double mutant mice at 6 and 24 hrs after i.t. *K. pneumoniae* administration. Bacterial challenge resulted in an early increase in total lung leukocytes in WT mice by 6 hrs, which was largely due to an increase in polymorphonuclear leukocytes (PMN). As compared to WT infected animals, TLR4^lps-d^ and TLR4^lps-d^/TLR9^−/−^ mice displayed significantly lower numbers of lung PMN at this time point (Table 1). By comparison, numbers of total leukocytes and PMN in infected TLR9^−/−^ mice did not significantly differ from similarly treated WT animals. By 24 hrs post *K. pneumoniae* administration, no differences in total leukocytes or PMN were observed in any of the groups examined. Additionally, no differences in numbers of lung monocyte/macrophages, CD4+ or CD8+ T cells was observed at 6 or 24 hrs in the four groups examined (Table 1 and data not shown).

**Table 1:** Cell recruitment after i.t. *Klebsiella* challenge in WT and mutant mice.

**Cell count**	**WT/Un**	**WT/kp**	**TLR4 ^lps-d^/kp**	**TLR9^−/−^/kp**	**TLR4 ^lps-d^9^−/−^/kp**
**Total cells**	1.2±0.2×106	24.6±2.2×106	24.2±1.3×106	25.5±1.3×106	24.2±2.5×106
**PMN**	0.4±0.73×106	8.2±1.32×106	3.1±1.01×106^*^	8.9±1.1×106	3.5±0.4×106^*^
**Monocytes/Macrophages**	0.8±0.4×106	15.2±2.06×106	21.2±2.06×106	17.7±2.03×106	21.5±3.03×106
**Total cells**	ND	32.3±1.3×106	33.1±3.5×106	31.2±3.3×106	33.3±4.4×106
**PMN**	ND	16.2±0.3×106	12.5±0.2×106	14.8±1.04×106	14.8±1.04×106
**Monocytes/Macrophages**	ND	16.1±2.03×106	20.2±2.34×106	16.6±1.15×106	19.5±1.05×106

Leukocytes were harvested from lung 6 and 24 hrs post *Klebsiella* administration by collagenase lung digest, and cytospins performed. *p<0.05 as compared to WT mice. n = 5 in each group. Un =  untreated, ND =  not done.

### Lung cytokine production in WT, TLR4^lps-d^, TLR9^−/−^ and TLR4^lps-d^/TLR9^−/−^ double mutant mice after *K. pneumoniae* administration

**Figure 3:**
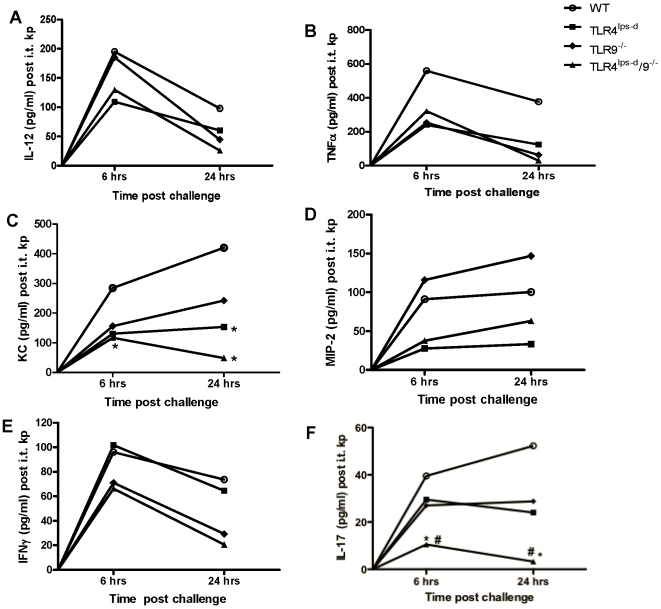
Cytokine production in whole lung after i.t. bacterial challenge. WT, TLR4^lps-d^, TLR9^−/−^, and TLR4^lps-d^/TLR9^−/−^ mice were challenged with 5×10^2^ CFU *Klebsiella*, then lungs harvested 6 hrs and 24 hrs post bacterial challenge. Cytokine levels were measured by ELISA. n = 5 in each group. *p<0.01 as compared to WT, # p<0.01 as compared to single mutant mice.

Tumor necrosis factor-alpha (TNF-α), chemokines, type 1 cytokines (IL-12, IFN-γ), and the Th17 cytokine IL-17 have been shown to be critically important cytokine mediators of innate antibacterial host responses in the lung [15]. To determine whether impaired localized expression of these cytokines could contribute to the increased bacterial burden observed in TLR single and double mutant mice, levels of TNF-α, KC/CXCL1, MIP-2/CXCL2, IL-12 p70, IFN-γ, and IL-17 were quantitated in lung homogenates by ELISA at 6 and 24 hrs post *K. pneumoniae* administration. As shown in Figure 3, bacterial administration to WT mice resulted in a rapid increase in the expression of all six cytokines by 6 hrs, with declining levels of TNF-α, IL-12 and IFN-γ at 24 hrs, while the expression of KC/CXCL1, MIP-2/CXCL2 and IL-17 continued to increase out to 24 hrs. The production of TNF-α was reduced in all mutant mice at 6 and 24 hrs. By comparison, the levels of IL-12 were decreased at both 6 and 24 hrs in TLR4^lps-d^, TLR4^lps-d^/TLR9^−/−^ mice, whereas late but not early IL-12 production was reduced in mice deficient in TLR9. KC/CXCL1 and MIP-2/CXCL2 production were reduced in both infected TLR4^lps-d^ and TLR4^lps-d^/TLR9^−/−^ mice, but not significantly affected in mice deficient in TLR9, compared to WT animals. IFN-γ production was significantly decreased in TLR9^−/−^ and TLR4^lps-d^/TLR9^−/−^ mice, but well maintained in TLR4 single mutant mice. As compared to infected WT mice, the production of IL-17 was moderately reduced in both TLR4^lps-d^ and TLR9^−/−^ mice (p<0.05), and was nearly completely extinguished in the lungs of TLR4^lps-d^/TLR9^−/−^ double mutant mice.

### IL-17 expression by CD4+ T cells and γδ T cells in WT, TLR4^lps-d^, TLR9^−/−^ and TLR4^lps-d^/TLR9^−/−^ double mutant mice after *K. pneumoniae* administration

**Figure 4:**
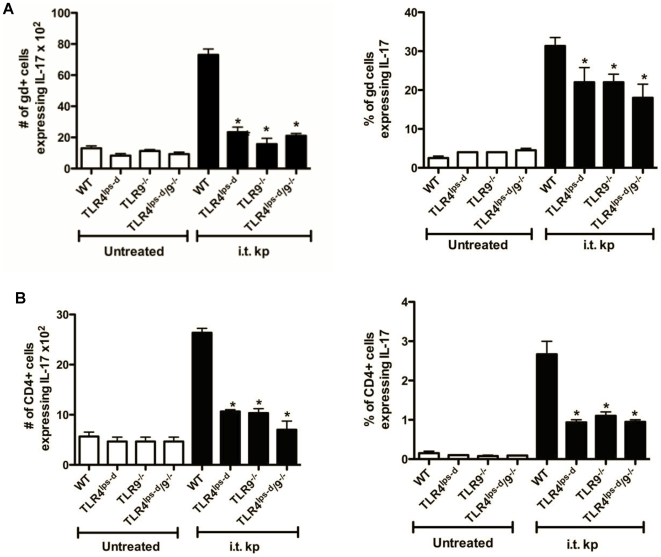
IL-17 expression by lung γδ and CD4+ T cells after i.t. *K. pneumoniae* administration. Flow cytometric analysis showing the number and percentage of γδ (A) and CD4+ T cells (B) with intracellular IL-17, 24 hours after i.t. *K. pneumoniae* challenge. n = 4 in each group. *p<0.05 as compared to WT post i.t. *K. pneumoniae*.

To determine which cells were responsible for reduced IL-17 production in mutant mice during bacterial pneumonia, we performed flow cytometry to quantitated the number and % of CD4+ T cells and γδ T cells expressing intracellular IL-17 in WT, TLR4^lps-d^, TLR9^−/−^ and TLR4^lps-d^/TLR9^−/−^ double mutant mice 24 hours post i.t. *Klebsiella* challenge. We focused on CD4+ T cells and γδ T cells, as these cells are believed to be the major cellular sources of IL-17 during lung bacterial infection [13], [16]. As shown in Figure 4, the % of CD4+ T cells expressing IL-17 was low in the uninfected state (<1%). In WT mice, there was a >10-fold increase in both the percentage and total number of cells co-expressing CD4 and IL-17. As compared to WT infected animals, the total number of CD4+/IL-17+ cells was decreased in infected TLR4^lps-d^, TLR9^−/−^ and TLR4^lps-d^/TLR9^−/−^ double mutant mice by 51, 56, and 68% respectively. The percentage of γδ T cells expressing IL-17 in WT infected mice was considerably higher than the percentage of CD4+ T cells. Similar to CD4+ T cells, the % and total number of IL-17+ γδ T cells was reduced in TLR4^lps-d^, TLR9^−/−^ and most notably TLR4^lps-d^/TLR9^−/−^ double mutant mice. (26, 24 and 21%, respectively). These findings indicate that both TLR4 and TLR9 contribute to IL-17 production from CD4+ T cells and γδT cells during bacterial pneumonia.

### IL-23 production in-vivo and from bone marrow derived dendritic cells (BMDC) isolated from WT and TLR mutant mice in-vitro in response to *K. pneumoniae*

**Figure 5:**
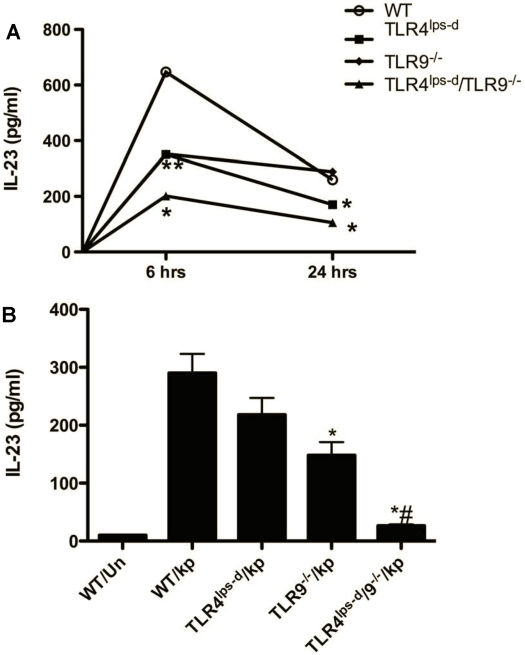
Expression of IL-23 in WT and mutant mice in-vivo and in-vitro. (A) Whole lung IL-23 production after i.t. *K. pneumonia* administration. n = 5 in each group. *p<0.05 as compared to WT post i.t. bacteria. (B) Secretion of IL-23 by BMDC incubated with heat killed *K. pneumoniae* for 18 hrs. n = 5 in each group. *p<0.01 as compared to WT BMDC; #p<0.05 as compared to single mutants.

The previous studies demonstrate reduced expression of IL-17 from TLR single and double mutant mice. As IL-23 is a strong endogenous inducer of IL-17 [13], [17], we next assessed the *K. pneumoniae*-induced expression of IL-23 in WT and mutant mice in-vivo and from BMDC in-vitro. As shown in Figure 5A, whole lung levels of IL-23 peaked in WT mice at 6 hrs post bacterial administration, returning toward baseline by 24 hrs. Maximum IL-23 production was significanly diminished in TLR4^lps-d^ and TLR9^−/−^ single mutant mice, and nearly completely mitigated in infected TLR4^lps-d^/TLR9^−/−^ double mutant mice. To determine if reduced IL-23 responses were attributable to impaired production of IL-23 by DC, we isolated BMDC from WT, single, and double mutant mice, incubated cells (5×10^5^/ml) with vehicle or heat killed *K. pneumoniae* (10∶1 MOI), then assessed for IL-23 secretion 16 hrs later. As compared to vehicle-exposed control cells, incubation with bacteria resulted in a 27-fold increase in IL-23 levels (Figure 5B). Importantly, production of IL-23 by *K. pneumoniae*-exposed BMDC isolated from TLR4^lps-d^ or TLR9^−/−^ mice was reduced by 31 and 48%, as compared to WT DC (p = 0.08 and <0.01, respectively). Moreover, IL-23 production by BMDC isolated from TLR4^lps-d^/TLR9^−/−^ double mutant mice was dramatically reduced, as compared to BMDC from WT and single mutant animals (p<0.05 for all groups).

### Ex-vivo lung macrophage phenotype in WT, TLR4^lps-d^, TLR9^−/−^ and TLR4^lps-d^/TLR9^−/−^ double mutant mice after *K. pneumoniae* administration

**Figure 6:**
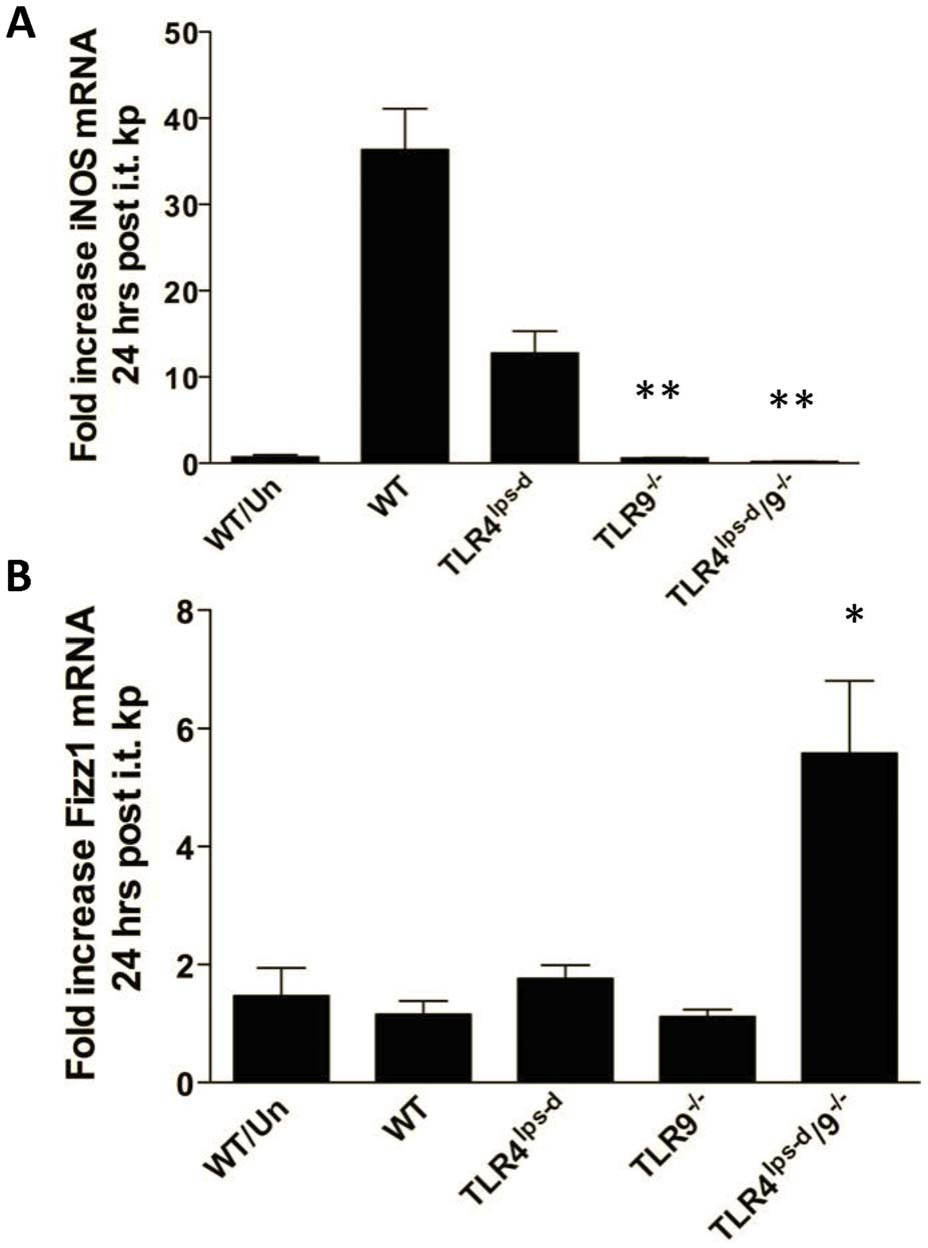
Expression of iNOS and FIZZ by alveolar macrophages. WT, TLR4^lps-d^, TLR9^−/−^, and TLR4^lps-d^/TLR9^−/−^ mice were challenged with 5×10^2^ CFU *Klebsiella* i.t. and BAL performed at 24 hours. Alveolar macrophages were harvested by adherence purification and iNOS and Fizz expression measured by RT-PCR. n = 5 in each group. *p<0.01 as compared to WT, †<0.05 as compared to TLR9^−/−^ mice.

We next assessed the activational status of lung macrophages isolated from WT and mutant mice 24 hrs after intrapulmonary bacterial challenge. The state of macrophage activation was determined by mRNA expression of iNOS as a marker of classical activation (M1) and Fizz-1 as a marker of alternative activation (M2). Alveolar macrophages were isolated from BAL ex-vivo by adherence purification, then constitutive expression of iNOS (NOS2) and Fizz-1 assessed by realtime quantitative PCR. As shown in Figure 6, *Klebsiella* infection in WT mice resulted in a marked upregulation of iNOS mRNA expression in lung macrophages (37-fold increase over uninfected controls). The expression of iNOS was partially reduced in lung macrophages from infected TLR4 single mutant mice, whereas the induction of iNOS was nearly completely mitigated in lung macrophages from TLR9^−/−^ and TLR4^lps-d^/TLR9^−/−^ mice. Interestingly, induction of Fizz-1 was detected only in alveolar macrophages isolated from infected TLR4^lps-d^/TLR9^−/−^ double mutant mice.

### Intrapulmonary administration of IL-17 improves lung and systemic bacterial clearance in TLR4^lps-d^/TLR9^−/−^ double mutant mice after *K. pneumoniae* challenge

**Figure 7:**
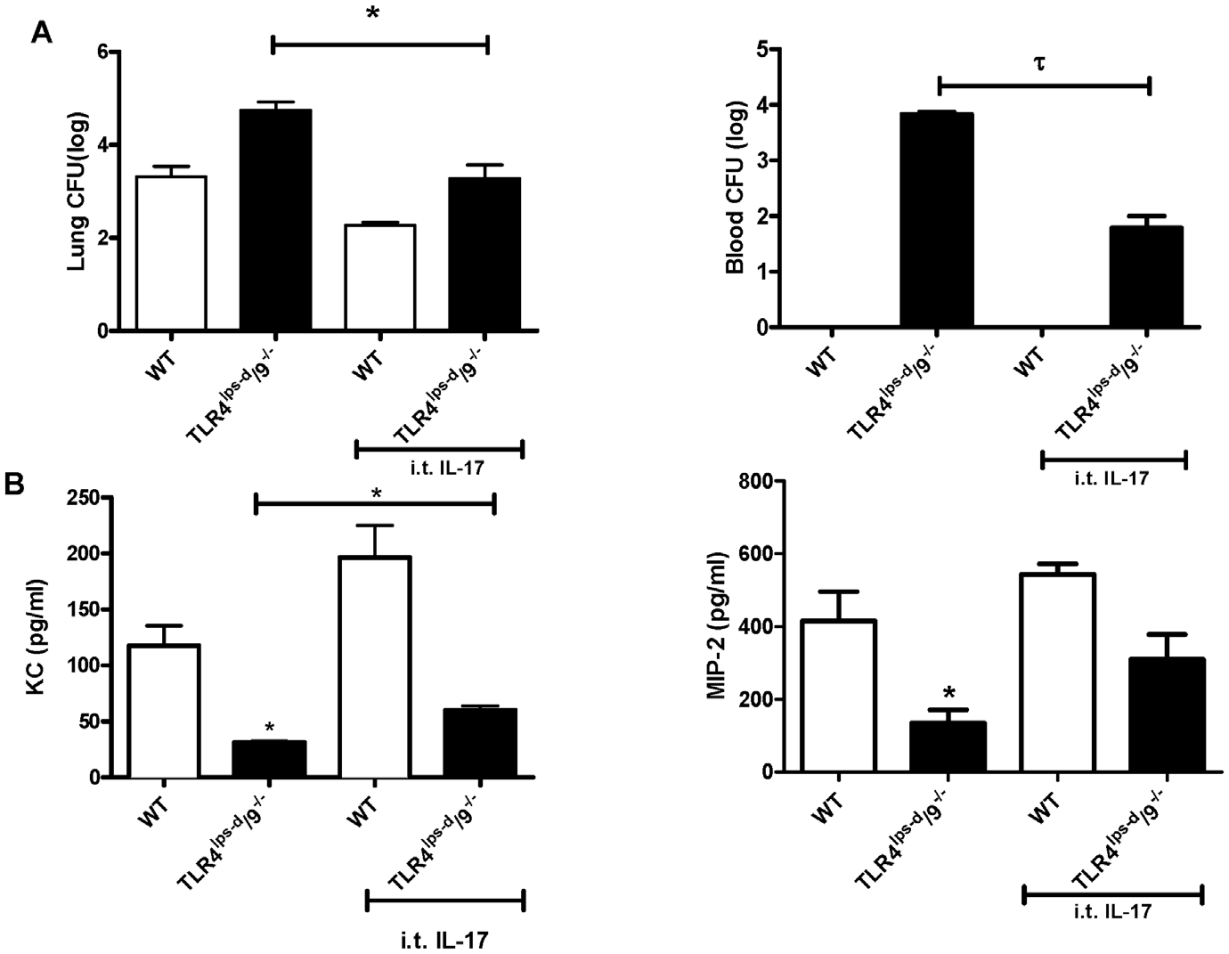
Bacterial clearance (A) and, lung expression of chemokines (B), in *Klebsiella*-infected WT and TLR4^lps-d^/TLR9^−/−^ mice post i.t. IL-17 treatment. WT or TLR4^lps-d^/TLR9^−/−^ mice were challenged with 8×10^2^ CFU *K. pneumoniae* followed immediately by intratracheal administration of 1 µg rm IL-17A or vehicle. (A) Blood and lungs were collected at 24 hours post challenge and CFU quantitated. (B) Whole lung KC/CXCL1 and MIP-2/CXCL2 levels in WT and TLR4^lps-d^/TLR9^−/−^ mice 24 hours post treatment with *K. pneumoniae*. n = 3−5 per group. *p<0.01 and τ<0.001.

To determine if the defect in IL-17 production contributed to impaired host defense against *K. pneumoniae* in the TLR4^lps-d^/TLR9^−/−^ double mutant mice, we performed rescue experiments using recombinant murine IL-17 administered i.t. immediately after i.t. *Klebsiella* challenge. Wildtype and TLR4^lps-d^/TLR9^−/−^ mice were administered 8×10^2^ CFU *Klebsiella* followed sequentially by i.t. rm IL-17A (1 µg) or vehicle, then blood and lungs harvested 24 hrs later. As compared to WT animals, vehicle-treated TLR4^lps-d^/TLR9^−/−^ mice displayed a significantly higher burden of *K. pneumoniae* in lung tissue and increased systemic dissemination, as measured by blood CFU (Figure 7A). Treatment with IL-17 in WT mice resulted in a 21-fold reduction in lung *K. pneumoniae* CFU. None of the WT mice developed bacteremia. However, treatment of TLR4^lps-d^/TLR9^−/−^ mice with IL-17 resulted in a more substantial 156- and 215-fold reduction of *K. pneumoniae* CFU in lung and blood, respectively (p<0.01 for lung and p<0.001 for blood). Interleukin-17 has been shown to induce neutrophil active CXC chemokines from lung macrophages in pneumonia [15], [18]. Interestingly, the i.t. administration of IL-17 resulted in a 2- and 2.2-fold induction of KC/CXCL1 and MIP-2/CXCL2 in infected TLR4^lps-d^/TLR9^−/−^ mice (Figure 7B, p<0.05 and p = 0.08, respectively), although treatment with IL-17 failed to restore lung chemokines levels to that observed in infected WT animals.

## Discussion

Toll like receptors are responsible for innate recognition of microbes. Previous studies have identified several TLRs, including TLR4, TLR5, and TLR9 as active participants in lung antibacterial immunity against extracellular Gram-negative bacterial pathogens [6], [8], [19]. While the contribution of individual TLRs have been well described, the temporal importance and potential interactions between TLRs during bacterial infection has not been thoroughly investigated. Our study indicates that TLR4 and TLR9 have both non-redundant and complementary functions during the generation of protective innate immunity. Moreover, we found that TLR4 and TLR9 regulate lung IL-23 and IL-17 responses in pneumonia.

Similar to previous reports, we observed impaired lung bacterial clearance in mice with defective TLR4 or deletion of TLR9 [8], [14], [20]. However, the greatest defect in lung bacterial clearance was observed in TLR4^lps-d^/TLR9^−/−^ double mutant mice, indicating that both TLR4 and TLR9 are required for optimal clearance. The early influx of PMN was markedly reduced in *Klebsiella*-infected TLR4^lps-d^ mice, which may be due to impaired production of the neutrophil active chemokines [21]. Moreover, TLR4 appears to drive the early production of the type 1 promoting cytokine IL-12. By comparison, defects in later production (24 hrs) of IL-12 and expression of the activating cytokine IFN-γ was observed in TLR9 deficient mice after bacterial challenge, consistent with the notion the TLR9 promotes type 1 immunity during pneumonia. Importantly, both TLR4 and TLR9 contribute to the production of TNF-α, IL-23 and IL-17, and maximal expression of IL-17 responses appears to require both of these TLRs.

Alterations in the lung cytokine milieu are a probable cause for differential activation of pulmonary macrophages in mutant mice during pneumonia. Impaired classical activation of macrophages (as manifest by constitutive ex-vivo expression of iNOS) was observed in cells from all mutant mouse strains post infection, but most prominent in macrophages with defective TLR9 signaling (either TLR9^−/−^ or TLR4^lps-d^/TLR9^−/−^ cells). This is consistent with our previous finding of impaired expression of iNOS and nitric oxide by lung macrophages from *Klebsiella*-challenged TLR9^−/−^ mice, which was associated with reduced intracellular bacterial killing but not phagocytic responses. IFN-γ is a key driver of classical macrophage activation [22], and the substantial impairment in the production of this cytokine in TLR9 single or double mutant mice corresponds with reduced iNOS expression. Interestingly, the expression of Fizz-1 as a marker of alternative activation or M2 phenotype [23], [24] was found only in macrophages from double mutant mice, suggesting that both TLR4 and TLR9 are required to prevent alternative macrophage activation during infection.

Differences in the expression of various cytokines in the TLR mutant mice may be accounted for by cell-specific expression of these TLRs. For example, lung macrophages express TLR4 but minimal TLR9 [25], which may contribute to early production of TNF-α and chemokines. Similarly, structural cells, including the alveolar epithelium, express chemokines in response to both PAMPs and host-derived cytokines elaborated by pulmonary macrophages [26], [27], [28], [29]. By comparison, TLR9 expressing dendritic cells elaborate type 1 promoting cytokines that drive the production of IFN-γ from NK cells and T cells [8],[30]. Our flow cytometry studies indicate that γδ-T cells, and to a lesser extent CD4+ Th17 cells, are important sources of IL-17 during bacterial pneumonia. Moreover, the production of IL-17 by these cells is regulated by both TLR4 and TLR9. We cannot exclude direct TLR stimulation of γδ T cells by microbial products, as these cells have been shown to respond in a TLR4 dependent fashion [15], [31], [32], [33]. However, our data indicates that impaired IL-17 production in TLR4 and TLR9 mutant mice may be attributable to reduced IL-23 expression by DC and possibly other proximal cells, as IL-23 is known to be a major paracrine inducer of IL-17 in bacterial pneumonia [13], [15], [16] and we observed substantial defects in lung IL-23 production from TLR4/9 double mutant mice compared to infected WT animals. Impairment in the elaboration of IL-17 in double mutant mice clearly contributes to altered host immunity, as treatment with IL-17 largely restored bacterial clearance mechanisms in TLR4^lps-d^/TLR9^−/−^ mice. While treatment with IL-17 resulted in some reconstitution of CXC chemokine production, it is likely that full restoration of chemokines was not achieved due to diminished TNF-α expression, which has been shown to be required for optimal IL-17 mediated induction of selected CXC chemokines [18].

Survival studies performed indicate that mortality in TLR4^lps-d^ single mutant and TLR4^lps-d^/TLR9^−/−^ double mutant mice was similar, despite more dramatic impairment in lung bacterial clearance in the double mutant mice as compared the TLR4^lps-d^ single mutant mice. This suggests that mortality in this model is not completely dependent on efficacy of lung bacterial clearance. One distinct possibility accounting for differences in mortality is divergent roles of TLRs in regulating the magnitude lung injury. To this end, we have found that TLR4^lps-d^ and TLR9^−/−^ mice display quite different patterns of lung injury in response to *K. pneumoniae* challenge, as TLR4^lps-d^ mice develop substantial alveolar leak (as measured by BAL albumin levels) as compared to infected WT animals, whereas TLR9^−/−^ mice tended to be protected against lung injury as compared to WT mice (data not shown). Our finding of enhanced lung injury in TLR4^lps-d^ mice is consistent with the finding of increased lung injury in TLR4 deficient mice in response to intrapulmonary bleomycin administration or hyperoxic exposure [34], [35]. The role of TLR9 in regulating lung injury responses has not been reported but is a focus of ongoing investigations in our laboratory. Increased lung injury in TLR4^lps-d^ single mutant mice may also account for increased systemic dissemination in these animals relative to lung bacterial burden.

Collectively our study shows for the first time that TLR4 and TLR9 have distinct time-dependent and interactive functions during the development of protective innate antibacterial immunity in the lung. Modulation of TLR-mediated responses may represent an important target of therapy in patients with severe bacterial infection of the respiratory tract.
